# Beyond efficacy in water containers: Temephos and household entomological indices in six studies between 2005 and 2013 in Managua, Nicaragua

**DOI:** 10.1186/s12889-017-4296-6

**Published:** 2017-05-30

**Authors:** Jorge Arosteguí, Josefina Coloma, Carlos Hernández-Alvarez, Harold Suazo-Laguna, Angel Balmaseda, Eva Harris, Neil Andersson, Robert J Ledogar

**Affiliations:** 1 CIET International, Managua, Nicaragua; 2 Division of Infectious Diseases and Vaccinology, School of Public Health, University of California, Berkeley, CA USA; 3 Centro Nacional de Diagnóstico y Referencia, Ministerio de Salud, Managua, Nicaragua; 4 Centro de Investigación de Enfermedades Tropicales, Universidad Autónoma de Guerrero, Acapulco, Guerrero Mexico; 5 Department of Family Medicine, McGill University, Montreal, Canada; 6 CIET International, New York, NY USA

**Keywords:** Temephos, Clusters, *Aedes aegypti*, Camino Verde, Dengue prevention

## Abstract

**Background:**

A cluster-randomized controlled trial of community mobilisation for dengue prevention in Mexico and Nicaragua reported, as a secondary finding, a higher risk of dengue virus infection in households where inspectors found temephos in water containers. Data from control sites in the preceding pilot study and the Nicaragua trial arm provided six time points (2005, 2006, 2007 and 2011, 2012, 2013) to examine potentially protective effects of temephos on entomological indices under every day conditions of the national vector control programme.

**Methods:**

Three household entomological indicators for *Aedes aegypti* breeding were Household Index, Households with pupae, and Pupae per Person. The primary exposure indicator at the six time points was temephos identified physically during the entomological inspection. A stricter criterion for exposure at four time points included households reporting temephos application during the last 30 days *and* temephos found on inspection. Using generalized linear mixed modelling with cluster as a random effect and temephos as a potential fixed effect, at each time point we examined possible determinants of lower entomological indicators.

**Results:**

Between 2005 and 2013, temephos exposure was not significantly associated with a reduction in any of the three entomological indices, whether or not the exposure indicator included timing of temephos application. In six of 18 multivariate models at the six time points, temephos exposure was associated with *higher* entomological indices; in these models, we could exclude any protective effect of temephos with 95% confidence.

**Conclusion:**

Our failure to demonstrate a significant protective association between temephos and entomological indices might be explained by several factors. These include ecological adaptability of the vector, resistance of *Aedes* to the pesticide, operational deficiencies of vector control programme, or a decrease in preventive actions by households resulting from a false sense of protection fostered by the centralized government programme using chemical agents. Whatever the explanation, the implication is that temephos affords less protection under routine field conditions than expected from its efficacy under experimental conditions.

**Trial registration:**

ISRCTN 27581154.

**Electronic supplementary material:**

The online version of this article (doi:10.1186/s12889-017-4296-6) contains supplementary material, which is available to authorized users.

## Background

Over the last two decades, *Aedes aegypti* mosquito control in many countries has relied on household visits by centrally-run vector programmes to eliminate immature vector forms by placing the organophosphate larvicide temephos in clean household water containers. In some places, ultra-low volume pesticide spraying complements temephos placement to control the adult mosquito. In a strategy laid out 20 years ago and followed since then to intensify the “war against *Aedes aegypti*” [1], temephos placement in household water stores was “the fundamental operation of the attack phase” of the programme. The World Health Organization promotes integrated vector management [2] and there are reports of successful experiences of community involvement [3–6], yet community participation in dengue control is mostly still secondary to chemical-based control strategies run by centralized vector control programmes.

The Nicaraguan government has made substantial efforts to control the *Aedes aegypti* vector of dengue virus and to mitigate the impact of dengue epidemics. As in nearly all other countries in tropical and subtropical regions of the world, however, the *Aedes aegypti* mosquito that carries dengue and other arboviruses of medical relevance, continues to gain ground. After two decades of temephos use in the country, a recent paediatric cohort study in Nicaragua found an incidence rate of 16.1 cases and 90.2 dengue virus infections per 1000 person-years in children aged 2–14 years of age [7]. Complicating the public health picture are multiple viral strains, the increasing severity of clinical cases, and the increasing costs incurred by governments and communities due to dengue infection.

The well-documented temephos resistance [8–15] combined with recent explosive epidemics of zika and chikungunya across Latin America suggest the vector is out of control, fuelling concern about reliance on temephos in dengue prevention. This has spurred a search for sustainable alternatives to pesticide-based vector control, through biological approaches [16, 17], community self-management [3, 4] or evidence-based communication strategies [5].

A (2004–2008) pilot study in Managua, Nicaragua, in coordination with the *Centro Nacional de Diagnóstico y Referencia* (CNDR) of the Nicaraguan Ministry of Health, CIET International, the University of California at Berkeley, and the Sustainable Sciences Institute, established the feasibility and acceptability of a pesticide free approach [18, 19]. The intervention engaged communities in dengue vector control activities through dialogue centred on local evidence and their own experience. Impact assessment found a high level of acceptability and feasibility, improvement of entomological indices, and reduced risk of dengue infection in children, indicated by the level of anti-dengue virus antibodies in saliva before and after the dengue season.

Based on this experience, a multi-centred cluster-randomized controlled trial (2010–2013) tested the added value of community engagement in Managua, Nicaragua, and the Mexican state of Guerrero [5]. The official vector control programmes continued in both intervention and control neighbourhoods. The trial demonstrated a decrease in recent dengue virus infection risk, fewer self-reported cases of dengue illness and a reduction in entomological indices [5].

This secondary analysis of data from control (non-intervention) neighbourhoods in the Nicaraguan feasibility study and Nicaraguan arm of the trial assessed the impact on household entomological indicators of temephos application by the National Vector Control Programme. The dengue control programme of the Ministry of Health carries out 4–6 cycles of temephos abatement annually in all municipalities of Managua, but the coverage and actual periodicity of application varies year to year due to multiple local factors. In addition, the government programme conducts spatial fumigation and educational activities about elimination of *Aedes* reproduction sites.

## Methods

*The Camino Verde trial,* a pragmatic parallel group cluster randomised controlled trial, involved a random sample of communities in Managua, the capital of Nicaragua, and three coastal regions in Guerrero State in the south of Mexico [5]. A total of 60 clusters in Nicaragua and 90 in Mexico included 85,182 residents in 18,838 households. The community mobilisation protocol began with community discussion of baseline results. Each intervention cluster adapted the basic intervention -- chemical-free prevention of mosquito reproduction -- to its own circumstances. All clusters continued the government-run dengue control programme. Primary outcomes *per protocol* were self-reported dengue cases, serological evidence of recent dengue virus infection in children, and conventional entomological indices.

### Six measurement points

Data came from this three linked cross-sectional studies in this trial (2011, 2012 and 2013), and another three during the pilot study (2005, 2006 and 2007). Two of the six measurements points were in the dry season (2011 and 2013) and four during the rainy season (2005, 2006, 2007, 2012). During these entomological surveys, qualified government personnel collected, classified and counted *Aedes aegypti* larvae and pupae from households.

### Inspections and analysis of specimens

Twelve-person field teams conducted the household interviews and entomological inspections. Entomological inspections used the standard protocols of the national programme for inspecting, collecting, transporting, identifying, counting and classifying immature *Aedes aegypti* specimens. Inspectors checked every water container using the appropriate instruments (net, pipette, bowl, magnifying glass, flashlight) to find larvae or pupae. They classified containers as: barrels or large tanks, buckets, washtubs, flowerpot plates, flowerpots, tyres, containers for non-storage use (bowls, water fountains, etc.), and items that had no clear household use (*calaches*). The government entomologists verified and classified the collected specimens of larvae and pupae. A container was considered positive when it contained one or more immature forms of *Aedes aegypti* in any stage, confirmed by the government entomologists. A household was considered positive when it had one or more positive containers.

### Exposure to temephos

At six measurement points, the temephos exposure indicator came from the observation of temephos in inspected water containers. This served for the principal analysis. In a supplementary analysis at four measurement points (2006, 2007, 2012 and 2013), exposure to temephos came from two variables: i) temephos identified at the time of the entomological inspection in at least one container in the household (yes/no), and ii) the report in the household questionnaire of the last temephos application within 30 days of the interview (data binomialised at 30 days). We excluded from the analysis households unable to respond about the timing of the temephos application visits -- 10 in 2006 (<1%), 15 in 2007 (<1%), 361 in 2012 (9%) and 403 in 2013 (10%).

### Entomological indicators

We derived three entomological indicators of the presence of immature forms of the *Aedes aegypti* mosquito: The number of larvae- or pupae-positive households per 100 inspected households (Household Index), the households where pupae were found (Households Positive for Pupae (HPP) and the number of pupae per person (PPP).

### Statistical analysis

The principal analysis of the main trial used cluster as the unit in an intention to treat analysis [5]; the unit of analysis of this secondary analysis was the household because exposure to temephos was not uniform within the clusters. Bivariate and then multivariate analysis evaluated impact on each entomological index, for each exposure measure in the context of other factors that might affect the outcome, derived from household responses to an administered questionnaire.

For the Household Index analysis, we dichotomized into households with positive containers and without positive containers. For the HPP analysis, the dichotomized version identified households with pupae and households without pupae. For the PPP analysis we converted this continuous variable into a binomial variable by categorizing households according to whether they were above or below the overall mean value for the PPP variable. Other variables potentially related to the entomological outcomes varied from year to year, depending on the contents of the household questionnaire. Table 1 shows the variables derived from responses to the household questionnaire in each of the six surveys. We included all those variables in bivariate analysis significantly related to the outcome at the 5% level in a generalized linear mixed model (GLMM), using cluster as a random effect and discarding the first 100 iterations. The analysis used the Zelig programme (logit.mixed) in R [20] through CIETmap, an open-source interface with the R statistical programming language. We report the odds ratio (OR) and adjusted odds ratio (aOR) from this analysis.

**Table 1:** Variables potentially related to entomological outcomes, derived from responses to the household questionnaire in the six surveys

Variable	Variable included in the questionnaire					
	2005	2006	2007	2011	2012	2013
Choice of manual search for larvae or spending C$5 per week on temephos	Included & retained					
Assumed knowledge of life cycle of mosquito through recognizing a larva when shown	Included & retained	Included & retained		Included & retained	Included & retained	
Perception of a lack of temephos (yes/no)	Included but not retained					
Perception that the community itself can control the mosquito (ye/no)	Included & retained		Included & retained	Included but not retained		Included & retained
Ability to identify community leadership (can/cannot identify)	Included but not retained		Included but not retained			
Perception of danger from dengue in the community (yes/no)		Included & retained				
Perception of danger from dengue in the household (yes/no)		Included but not retained				
Discussion of dengue prevention with neighbours (yes/no)		Included & retained	Included & retained			Included & retained
Education of the head of the family (primary or less/above primary)					Included & retained	
Regularity of local water supply (regular/irregular)						Included & retained
Participation in local organisations (yes/no)						Included but not retained
Participation in a local dengue prevention activity (yes/no)						Included but not retained

“Included & retained” means the variable was included in the initial saturated GLMM model and retained in the final model.

“Included but not retained” means the variable was included in the initial saturated GLMM model but was not retained in the final model of variables all associated with the outcome at the 5% significance level.

## Results

Across the six time points, field teams made 20,869 entomological inspection visits in control neighbourhoods: 8564 in the three surveys during the pilot study and 12,305 in the three measurements of the trial itself. The six evaluations inspected 100,259 containers of which 8748 contained larvae or pupae; evaluators counted a total of 27,109 pupae (Table 2).

**Table 2:** Presence of *Aedes aegypti* larvae and pupae in surveys in six Managua control sites 2005 and 2013

Survey date	Number of clusters	Number of people	Number of households	Number (%) households with larvae or pupae	Number of containers	Number (%) containers with larvae or pupae	Number pupae observed
Oct 2005	20	15,619	2636	864 (33)	16,744	1441 (9)	4962
Oct 2006	20	15,561	2636	601 (23)	17,429	925 (5)	3306
Oct 2007	20	20,514	3556	818 (23)	15,337	1439 (9)	6014
Jan 2011	30	20,971	4031	750 (19)	18,276	911 (5)	2156
Aug 2012	30	21,666	4200	1499 (36)	16,732	2730 (16)	7576
Jan 2013	30	21,136	4064	897 (22)	15,741	1302 (8)	3095

Table 3 shows the association between the presence of at least one larvae or pupae positive container in the household and observed temephos presence, in six surveys in the Managua control clusters between 2005 and 2013; none of the six GLMM models showed a significant negative association between temephos presence and household positivity for larvae or pupae. Across the same six time points, Table 4 shows the association between pupae-positive households and temephos observed on inspection, and Table 5 shows the association between an above average PPP and temephos presence; again, none of the GLMM models at the six time points showed a significant negative association between temephos presence and the entomological outcome. In fact, six of the 18 models in Tables 3, 4 and 5 showed that the observed temephos presence was associated with a significant *increase* in the entomological index. The 95% confidence intervals of the OR in these nine models effectively exclude any protective effect of temephos. Additional file 1: Table S1-S3 show the findings when the analysis was repeated using the more stringent exposure indicator of temephos found upon inspection and reported to have been applied within the last 30 days. This was possible in four of the six surveys. Additional file 1: Table S1 shows the association between the presence of at least one larvae or pupae positive container in the household and temephos exposure; none of the four GLMM models showed a significant negative association between temephos exposure and household positivity for larvae or pupae. Additional file 1: Table S2 and S3 show associations between temephos exposure and pupae-positive households and pupae per person; again, none of the GLMM models at the four time points showed a significant negative association between temephos presence and the entomological outcome.

**Table 3:** Households with larvae or pupae positive containers (Household index) and temephos presence in surveys in Managua control sites between 2005 and 2013

Survey date	Percentage of households with temephos observed in any container	Temephos observed in any container		Temephos not observed in any container		OR (95% CI)^a^
		No. of inspected households	No. (%) with any positive containers	No. of inspected households	No. (%) with any positive containers	
Oct 2005	13.1	346	128 (37)	2290	736 (32)	1.28 (1.00–1.64)
Oct 2006	25.1	644	171 (27)	1992	430 (22)	1.18 (0.95–1.48)
Oct 2007	19.8	703	171 (24)	2853	647 (23)	0.97 (0.79–1.19)
Jan 2011	20.6	829	186 (22)	3202	564 (18)	*1.27 (1.04–1.55)*
Aug 2012	33.2	1394	562 (40)	2806	937 (33)	*1.45 (1.25–1.68)*
Jan 2013	23.7	966	298 (31)	3108	599 (19)	*1.82 (1.53–2.17)*

OR of >1.0 indicates that households with temephos present were *more* likely to have a positive entomological indicator; italic font indicates the association was significant at the 5% level

^a^Odds ratio and 95% confidence intervals from GLMM, with cluster as random effect

**Table 4:** Pupae-positive households and temephos presence in surveys in Managua control sites between 2005 and 2013

Survey date	Percentage of households with temephos observed in any container	Temephos observed in any container		Temephos not observed in any container		OR (95% CI)^a^
		No. of inspected households	No. (%) with any pupae	No. of inspected households	No. (%) with any pupae	
Oct 2005	13.1	346	73 (21)	2290	439 (19)	1.15 (0.86–1.54)
Oct 2006	25.1	644	69 (11)	1992	195 (10)	1.17 (0.86–1.59)
Oct 2007	19.8	703	98 (14)	2853	347 (12)	1.11 (0.85–1.43)
Jan 2011	20.6	829	70 (8)	3202	205 (6)	1.31 (0.98–1.76)
Aug 2012	33.2	1394	286 (21)	2806	539 (19)	1.17 (0.98–1.40)
Jan 2013	23.7	966	127 (13)	3108	259 (8)	*1.67 (1.31–2.13)*

OR of >1.0 indicates that households with temephos present were *more* likely to have a positive entomological indicator; italic font indicates the association was significant at the 5% level

^a^Odds ratio and 95% confidence intervals from GLMM, with cluster as random effect

**Table 5:** Number of people, number of pupae, and temephos presence in surveys in Managua control sites between 2005 and 2013

Survey date	Temephos observed in any container		Temephos not observed in any container		OR (95% CI)^a^
	No. people	No. pupae (PPP)	No. people	No. pupae (PPP)	
Oct 2005	2100	666 (0.3)	13,519	4296 (0.3)	1.18 (0.85–1.64)
Oct 2006	3736	955 (0.3)	11,825	2351 (0.2)	1.17 (0.83–1.66)
Oct 2007	3997	1271 (0.3)	16,517	4743 (0.3)	1.01 (0.74–1.37)
Jan 2011	4378	404 (0.1)	16,592	1751 (0.1)	1.31 (0.97–1.75)
Aug 2012	7321	2896 (0.7)	14,345	4680 (0.3)	*1.36 (1.11–1.67)*
Jan 2013	5086	1216 (0.2)	16,052	1879 (0.1)	*1.83 (1.41–2.37)*

We dichotomised the pupae per person (PPP) variable into households with above and below the mean PPP

OR of >1.0 indicates that households with temephos present were *more* likely to have a positive entomological indicator; italic font indicates the association was significant at the 5% level

^a^Odds ratio and 95% confidence intervals from GLMM, with cluster as random effect

In households reporting temephos application in the last 30 days, entomological inspectors observed the larvicide in at least one container in less than 40% (3815/9604). There were differences in the proportion of barrels and washtubs with larvicide at the time of the inspection. Inspectors detected temephos in one third of barrels: 42% in 2012 (1152 / 2760) and 35% in 2013 (917 / 2631). They found it in less than 15% of washtubs: 10% in 2006 (466 / 4467), 7% in 2007 (459 / 6274), 14% in 2012 (623/4335) and 7% in 2013 (294/4326).

It was possible in the three trial-related surveys to consider individual temephos-treatable containers that were positive to *Aedes* larvae or pupae. In 2011, inspectors found 7% of containers held temephos, in 9% of which they also found larvae or pupae (115/1293); in 2012, 16% held temephos of which 21% also had larvae or pupae (495/2408); in 2013, 9% held temephos of which 19% also had larvae or pupae (276/1480).

## Discussion

Across the six surveys included by this study between 2005 and 2013, we could not confirm a single statistically significant association between presence of temephos and reduction in household entomological indices. Whatever the efficacy of temephos in individual containers [21], this result is compatible with temephos failing to reduce the development stages of *Aedes aegypti* in households in the real life setting. There are several possible explanations.

### Chemical resistance

The first evidence of temephos resistance was documented in Cuba in 1999 [8], and this has been followed by reports of resistance from several other Latin American countries [9–14] To date there has been no conclusive documentation in Nicaragua of *Aedes aegypti* resistance to temephos. Reports on resistance from other countries suggest this is possible. Our finding of larvae or pupae in around 20% of temephos treated containers indicates resistance is quite likely.

### Supply deficiencies

It is also possible that consignments of temephos acquired and used by the government programme during the study years were defective. This would not be unique in the history of pesticide, suggesting purchasers should exercise more careful quality control and supervision of the storage and distribution of the product [22].

### Quality and coverage of temephos application

It is possible that the official programme failed to cover all households, failed to include all potential breeding sites within the household, failed to apply the product in the proper concentration for each receptacle, or failed to distribute the product with the required frequency. Complying with the stringent norms for the larvicide application [2] is a challenge for any health authority: a study in two cities in Nicaragua showed that in order to control pupae effectively temephos needs to be applied at least every 30 days [23]. Across the surveys reported here, on average about two of every three households reported that they had received a temephos application visit in the previous 30 days.

### Use of domestic water containers

In the four surveys in which we asked about the timing of the temephos visits, entomological inspections found the larvicide in only 24–37% of households that reported temephos had been distributed to them during the previous month. Official norms require larvicide to be placed in all containers used for storing water [24]. Both washtubs and tanks are targeted for temephos but people are more likely to empty washtubs needed for washing or laundry. This is borne out by the finding of temephos in fewer washtubs (15%) than tanks (42%). The alternative uses of temephos-treated washtubs, beyond storing water, reduce pesticide persistence and therefore the value of the temephos strategy [25]. Frequent topping up of storage tanks also reduces temephos effectiveness [26].

### “false sense of security”

The main report of the Camino Verde trial [5] suggested the extensive government effort to distribute temephos might have been a disincentive to manual approaches to vector control. This explanation emerged from post-trial focus group discussions and the result from the trial indicating that in households where inspectors found temephos in water containers, residents were less likely to report participation in community activities to prevent dengue [5]. If temephos is only partially effective but its presence reduces other vector control efforts by households, such as cleaning and covering water containers, temephos presence could be associated with a higher risk of positive entomological indicators.

### Mosquito behaviour

Variations in the mosquito’s choice egg-laying sites from year to year might explain the failure of temephos to reduce entomological indices in some years more than others [27, 28].

We are aware of the limitations of our study, a secondary analysis of data from a study not designed to investigate temephos effectiveness. As a series of cross-sectional studies, our results have the usual limitations with regard to causality inference. Limiting the analysis to households in the control neighbourhoods allowed us to exclude the effect of the *Camino Verde* intervention, and to reflect the general vector control conditions in the region. We do not anticipate recall bias in household reports of temephos placements since respondents were not aware of the entomological findings at the time of interview. The proportion of households that did not provide an answer to the question about the timing of the last temephos visit was much lower in the earlier surveys (less than 1% in 2006 and 2007, compared with around 10% in 2012 and 2013), implying a change in quality of administration of the questionnaires. Again, this was independent of the entomological findings, and does not explain the consistent lack of protective associations with temephos across the time points. Dichotomizing variables like pupae per person can lose information, especially as we are detecting a negative result. Our household pupae variable (pupae positive household or PPH) implies zero pupae vs any pupae. The question is whether temephos can reduce the pupal count while not changing, or sometimes while changing for the worse, the proportion of pupae-free households. This seems implausible.

Our findings contradict a recent systematic review of studies of the impact of temephos, used alone or in combination with other interventions. Although with very few randomised controlled trials, the review reported a reduction in vector indices in 11 studies (four before-after and seven non-randomised trials) that measured the efficacy of temephos as a single intervention, mostly compared with no intervention [29]. The review concluded the effect of temephos on vector indices, when applied as part of a complex intervention, was much less convincing [29]. Outside the research context, *Aedes aegypti* control almost everywhere implies combined interventions and often unpredictable cluster dynamics [30]. Using temephos found physically upon inspection as the measure of temephos exposure, our study was concerned with the observable effectiveness of a real-life temephos programme, rather than measuring the efficacy of specifically applying temephos, with or without other interventions.

## Conclusion

We found no evidence to support the idea that temephos placed in household water containers by a government vector control programme reduces conventional entomological indices. In one third (6/18) of GLMM models at six time points, however, temephos was a significant risk factor for higher entomological indices; these models could *exclude* a protective temephos effect with 95% confidence. Our finding of larvae or pupae in around 20% of temephos treated containers indicates resistance is a likely if partial explanation.

While temephos might work in a research setting as a single intervention compared with no intervention, we found no evidence of a protective effect in a community setting under the non-experimental conditions of a national programme. Whether this is due to defective lots of pesticide, resistance, application procedures or a combination of these, temephos seems to be largely irrelevant to *Aedes aegypti* control. Year to year differences in the control programme could be associated with differing levels of demotivation or a false sense of security. Thus, in some years but not others, temephos was actually a measurable risk factor.

## Additional file

Additional file 1: Tables of analysis of entomological indices and temephos exposure confirmed by both observation and reported recent application. (PDF 20 kb)

## Abbreviations

CNDR: National Diagnostics and Reference Centre (Ministry of Health)
CRCT: Cluster-randomized controlled trial
DENV: Dengue virus
NVCP: National Vector Control Programme
