# Supplementary material for: Beyond efficacy in water containers: Temephos and household entomological indices in six studies between 2005 and 2013 in Managua, Nicaragua
Source: BMC Public Health. 2017 May 30;17(Suppl 1):434. doi: 10.1186/s12889-017-4296-6 (PMC5506593; doi:10.1186/s12889-017-4296-6)
Supplement: Additional file 1: — Tables of analysis of entomological indices and temephos exposure confirmed by both observation and reported recent application. (PDF 20 kb) [file 12889_2017_4296_MOESM1_ESM.pdf]

Table S1. Households with any larvae or pupae positive containers (Household index) and temephos presence and timing of application in four surveys in Managua control sites between 2006 and 2013

| Survey date | Temephos present *and* temephos visit within the last 30 days | | No temephos present, or temephos visit more than 30 days ago, or no visit | | OR (95% CI)* |
|---|---|---|---|---|---|
| | No. inspected households | No. (%) with positive containers | No. inspected households | No. (%) with positive containers | |
| Oct 2006 | 549 | 140 (25) | 2,085 | 461 (22) | 1.07 (0.84-1.35) |
| Oct 2007 | 579 | 125 (22) | 2,965 | 690 (23) | 0.80 (0.64-1.01) |
| Aug 2012 | 1,034 | 379 (37) | 1,049 | 421 (40) | 1.06 (0.87-1.30) |
| Jan 2013 | 599 | 161 (27) | 3,072 | 641 (21) | **1.35 (1.09-1.67)** |

* Odds ratio and 95% confidence intervals from GLMM, with cluster as random effect.

OR of >1.0 indicates that households with temephos present and applied within last 30 days were *more* likely to have a positive entomological indicator; bold font indicates the association was significant at the 5% level

Table S2. Pupae-positive households and temephos presence and timing of application in four surveys in Managua control sites between 2006 and 2013

| Survey date | Temephos present *and* temephos visit within the last 30 days | | No temephos present, or temephos visit more than 30 days ago, or no visit | | OR (95% CI)* |
|---|---|---|---|---|---|
| | No. inspected households | No. (%) with pupae | No. inspected households | No. (%) with pupae | |
| Oct 2006 | 549 | 59 (11) | 2,085 | 205 (10) | 1.18 (0.85-1.63) |
| Oct 2007 | 579 | 71 (12) | 2,965 | 373 (13) | 0.92 (0.69-1.23) |
| Aug 2012 | 1,034 | 185 (18) | 1,049 | 245 (23) | 0.87 (0.70-1.11) |
| Jan 2013 | 599 | 68 (11) | 3,072 | 280 (9) | 1.32 (0.98-1.77) |

* Odds ratio and 95% confidence intervals from GLMM, with cluster as random effect.

OR of >1.0 indicates that households with temephos present and applied within the last 30 days were *more* likely to have a positive entomological indicator; bold font indicates the association was significant at the 5% level

Table S3. Number of people, number of pupae, and temephos presence and timing of application in four surveys in Managua control sites between 2006 and 2013

| Survey date | Temephos observed in any container | | | Temephos not observed in any container | | | OR (95% CI)* |
|---|---|---|---|---|---|---|---|
| | No. households | No. people | No. pupae (PPP) | No. households | No. people | No. pupae (PPP) | |
| Oct 2006 | 549 | 3,207 | 881 (0,3) | 2,085 | 12,343 | 2,425 (0.2) | 1.21 (0.84-1.74) |
| Oct 2007 | 579 | 3,292 | 773 (0.2) | 2,965 | 17,160 | 5,232 (0.3) | 0.74 (0.51-1.05) |
| Aug 2012 | 1,034 | 5,449 | 1,790 (0.3) | 1,049 | 5,375 | 2,351 (0.4) | 0.83 (0.63-1.09) |
| Jan 2013 | 599 | 3,200 | 672 (0.2) | 3,072 | 15.924 | 1,985 (0.1) | **1.50 (1.10-2.06)** |

* Odds ratio and 95% confidence intervals from GLMM, with cluster as random effect.

We dichotomised the pupae per person (PPP) variable into households with above and below the mean PPP.

OR of >1.0 indicates that households with temephos present and applied within the last 30 days were *more* likely to have a positive entomological indicator; bold font indicates the association was significant at the 5% level
